# Cruciate retaining total knee arthroplasty has a better 10 year survival than posterior stabilized total knee arthroplasty: a systematic review and meta-analysis

**DOI:** 10.1186/s40634-023-00583-2

**Published:** 2023-02-17

**Authors:** Raj Kanna, S. M. Murali, Ashok Thudukuchi Ramanathan, Lester Pereira, C. S. Yadav, Sumit Anand

**Affiliations:** 1 Associate Professor in Orthopaedics, Madha Medical College and Research Institute, Chennai, Tamil Nadu India; 2 Associate Profesoor in Orthopaedics, SMMCH&RI Chennai, 600 069, Tamil Nadu India; 3 Associate Professor in Orthopaedics, Sri Ramchandran Institute of Higher Education and Research, Chennai, India; 4 Department of Orthopaedics, Primus Hospital, Delhi, India

**Keywords:** Total Knee Arthroplasty, Cruciate retaining, Posterior stabilized, Survivorship, Complications

## Abstract

**Purpose:**

There has been a long standing debate regarding superiority of cruciate retaining total knee arthroplasty over posterior stabilized total knee arthroplasty regarding the short-term outcomes as well as long-term survivorship. The proponents of both the techniques have published vast evidence in favor of their respective surgical method and early outcome in meta-analyses does not seem to be significantly different. The decision to select either design should depend on their long-term survivorship but the literature comparing their long-term survival is sparse.This meta-analysis was conducted in order to answer the following questions: (1) Does cruciate retaining total knee arthroplasty has a better long-term survival beyond 10 years.compared to posterior stabilized total knee arthroplasty? (2) Does cruciate retaining knee arthroplasty has higher complication rates compared to posterior stabilized total knee arthroplasty?

**Methods:**

The present systematic review and meta-analysis study was carried out following PRISMA guidelines. The following databases: Embase, Web of Science, PubMed, Scopus, the Cochrane Library, Google Scholar, and CINAHL were used to search potentially interesting articles published from database inception until January 2022. Inclusion criteria for articles were: (1) retrospective comparative studies; (2) patients who had undergone a total knee arthroplasty; (3) publications evaluating the long-term survival of cruciate-retaining (CR) versus posterior stabilizing (PS) at a minimum 10 years’ follow-up; (4) publications evaluating complications of cruciate-retaining (CR) versus posterior stabilizing (PS) at a minimum 10 years’ follow-up; and (5) publications reporting sufficient data regarding the outcomes. We used a fixed-effects design in the case of I^2^ < 50% and *P* > 0.05; if not, we adopted a random-effects design [4]. We also performed subgroups and sensitivity analysis in order to assess the possible source of heterogeneity.

**Results:**

Database searching identified 597 studies to be screened, of which 291 abstracts were revealed as potentially eligible and finally 7 articles were included. The forest plot showed that CR had significantly better survival than PS (OR = 2.17; 95% CI: 1.69–2.80) after 10 years. However, complication rate was not significantly different between CR and PS groups (OR = 0.86; 95% CI: 0.52–1.44; *P* = 0.57). Subgroup analysis showed that only the period of publication constituted a source of heterogeneity in survivorship outcome. Sensitivity analysis revealed that outcomes did not differ markedly, which indicates that the meta-analysis had strong reliability.

**Conclusion:**

The results of this meta-analysis showed that cruciate retaining prosthesis may be preferred over the posterior stabilized design in view of longer survivorship it offers However, further randomized controlled trials are recommended to confirm this finding.

## Introduction

The knee arthroplasty surgeons over the world are conceptually divided on whether to retain the cruciate ligament [1]. The supremacy debate between cruciate retaining (CR) and posterior stabilized (PS) total knee arthroplasty (TKA) has now entered into its fourth decade and does not seem to conclude in near future [2, 3]. Though literature comparing outcomes of CR and PS TKA has accumulated over the years, we are far from a consensus. Some studies claim that there is no difference between the two designs in terms of clinical outcome [2], others find that PS TKA results in better range of motion [4, 5] and few others claim that CR TKA has better proprioception [6]. In general, PS TKA design is considered to be a better option for knees with severe deformity [7, 8]. The advocates of PS TKA report that substituting PCL with a tibial post improves femoral rollback and results in better range of motion [9]. They also claim that the cruciate retention leads to paradoxical rollback which decreases the knee flexion [10]. However, CR TKA surgeons find that the resultant decrease in flexion is not of clinical significance as their patients have better outcome scores [11]. CR design confers better stability and better proprioception, required for demanding activities like kneeling and climbing stairs [11, 12]. Considering the fact that PS and CR TKA fare equally at short-term and mid-term [13], the decision to select either design should depend on their long-term survivorship. The literature comparing long-term survival of designs is sparse. Though Registry data from many countries is in favour of cruciate retaining TKA [14, 15], this meta-analysis was done to generate higher level evidence for guiding surgical practices. Our research hypothesis was: Both cruciate retaining and posterior stabilized total knee arthroplasty have (1) similar long-term survival beyond 10 years (2) similar complication rates.

## Methods

### Search strategy and criteria

The present systematic review and meta-analysis study was carried out following PRISMA guidelines. The following databases: Embase, Web of Science, PubMed, Scopus, the Cochrane Library, Google Scholar, and CINAHL were used to search potentially interesting articles published from database inception until January 2022. A systematic search was conducted by 2 independent reviewers, using the following terms “survival” OR “overall survival” OR “survivorship” AND “posterior cruciate-retaining” OR “cruciate-retention” OR “cruciate-retaining” OR “minimally stabilized” OR “unconstrained” AND “posterior cruciate-stabilizing” OR “posterior-stabilized” OR “posterior cruciate-substituting” OR “posterior substituting”.

### Inclusion and exclusion

Relevant articles were screened by title and abstract after suppression of duplicates. Studies were eligible for inclusion if they addressed the long-term survival of cruciate-retaining (CR) versus posterior stabilizing (PS) in total knee arthroplasty (TKA) at a minimum 10 years’ follow-up. The remaining studies were then examined in full text to confirm eligibility. In addition, the reference lists of all the included articles were manually examined to identify eligible reports that might have been missed in the initial search.

Inclusion criteria for articles were: (1) retrospective comparative studies; (2) patients who had undergone a total knee arthroplasty; (3) publications evaluating the long-term survival of cruciate-retaining (CR) versus posterior stabilizing (PS) at a minimum 10 years’ follow-up; (4) publications evaluating complications of cruciate-retaining (CR) versus posterior stabilizing (PS) at a minimum 10 years’ follow-up; and (5) publications reporting sufficient data regarding the outcomes. Exclusion criteria for studies were: (1) no full text electronically available; (2) publications in a language other than English, (3) letters, editorials, comments, protocols, review papers, and guidelines; and (4) articles with limited outcome information.

All retrieval processes were performed independently by two researchers and discordances were solved by discussion.

### Assessment of study quality

The quality of each article was appraised by two individual reviewers using the ROBINS 1 assessment tool for non-randomized control trials [16]. The ROBINS 1 tool assesses seven potential sources of bias including bias due to confounding, bias in selection of participants into the study, bias in classification of interventions, bias due to deviations from intended interventions, bias due to missing data, bias in measurement of outcomes, and bias in selection of the reported results.

### Data collection and abstraction

Two independent authors retrieved information from the eligible articles following the inclusion and exclusion criteria, and information were collected on a standardized data sheet that included author name, year, type of study, geographic origin, sample size, sex and age of participants, and outcome ascertainment.

### Other methods

#### Outcome measure

The primary outcome was the long-term survival and the secondary outcomes were complications (instability, infection,fracture etc.) and failure rates of cruciate-retaining (CR) versus posterior stabilizing (PS) in total knee arthroplasty (TKA) at a minimum 10 years’ follow-up.

### Statistical analyses

RevMan V5.4 (Cochrane Collaboration, Oxford, United Kingdom) was used to conduct the statistical analyses. Mantel–Haenszel method was used to calculate Odds Ratio (OR) with 95% confidence intervals in order to evaluate the different outcomes. A value of *P* < 0.05 was considered as the level of significance. The Cochrane chi-squared test was conducted to evaluate heterogeneity among articles, with *P* value < 0.05 indicating the existence of heterogeneity. To estimate the impact of heterogeneity on the meta-analysis, I2 value was calculated. Indeed, I^2^ values ≥ 50% and *P* < 0.05 indicated a moderate to high degree of heterogeneity among pooled studies. A fixed-effects design was used in the case of I^2^ < 50% and *P* > 0.05; if not, a random-effects design was adopted [17]. We also performed sensitivity analysis in order to assess the possible source of heterogeneity.

## Results

### Results from search, screening and final inclusion

**Fig. 1:**
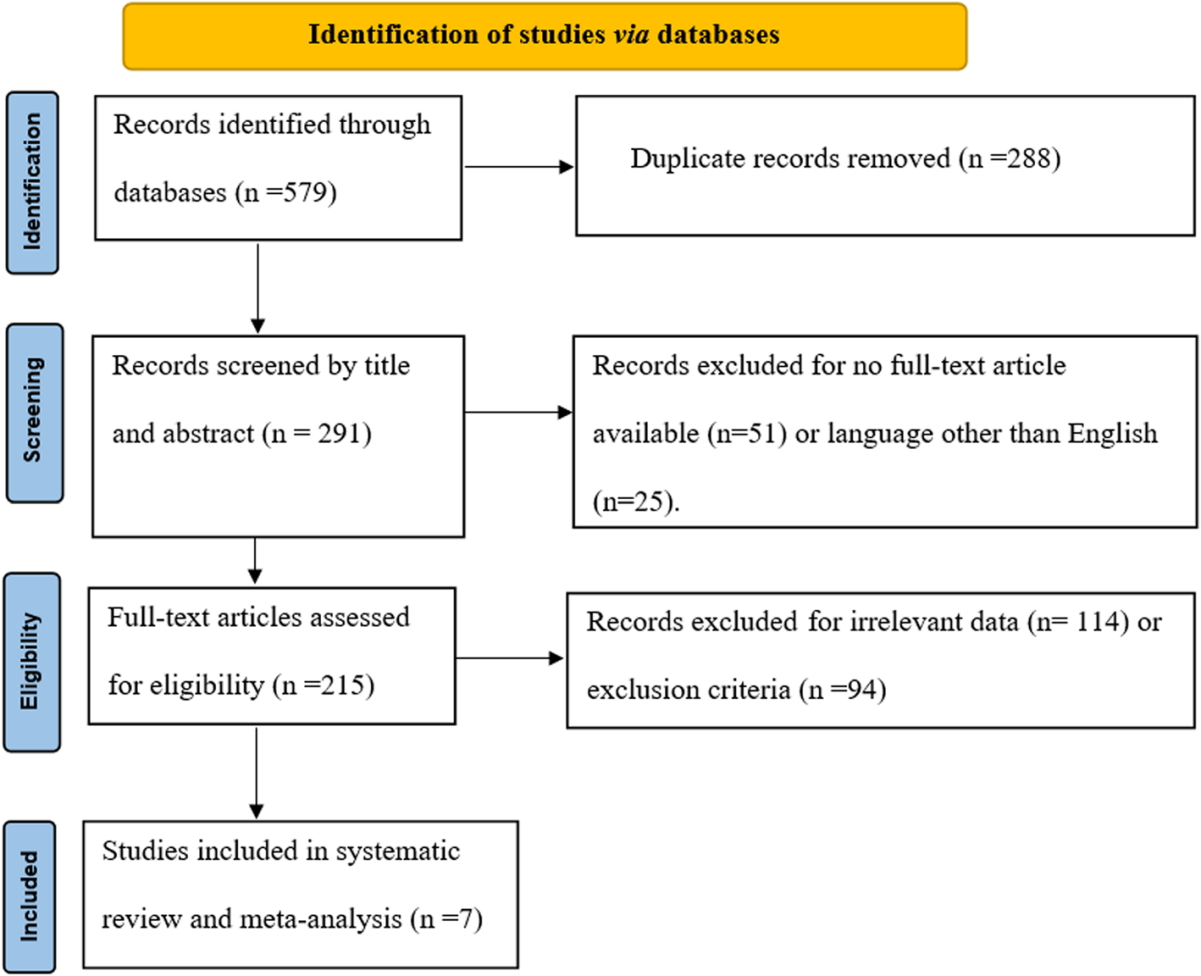
An outline of the PRISMA instructions used to carry out this systematic review and meta-analysis

Database searching identified 597 studies to be screened, of which 291 abstracts were revealed as potentially eligible and retrieved for full text review. Eligibility criteria were met by 7 articles, which were belonged to this systematic review and meta-analysis study. PRISMA study flowchart is presented in Fig. 1.

The included articles were issued between 2003 and 2021. Three studies were conducted in USA, while the remaining ones were performed in France, Spain, UK and Republic of Korea. The sample size of the included articles varied from 99 to 8290 and the total number of patients was 10,140. In summary, the total number of TKA was 21 399 with 14 189 CR and 6 650 PS. The follow-up period varied between 5 and 20 years. All the included studies had a level of evidence = 3. Studies’ characteristics are summarized in Table 1.

**Table 1:** Studies’ characteristics

Article, year	Country	Study design	Period	Number of patients	Sex	Mean age	Total knee arthroplasties	CR	PS	Survival Follow-up	Overall survival	
											CR	PS
Abdel et al. 2011 [18]	USA	Retrospective study	1988 to 1998	ND	CR group: 54% women and 46% men PS group: 56% women and 43% men	CR group: 70 years PS group: 68 years	8117	5389	2728	5, 10, 15 Years	- 10 year:95% - 15 years: 88%	- 10 year:90% - 15 years: 75%
Argenson et al. 2013 [9]	France	Retrospective study	The year 2000	828	274 men 554 women	71 years	609	139	470	10 years	- fixed CR:95% -mobile CR:91%	- fixed PS:90%—mobile PS:94%
Kim et al. 2021 [19]	Republic of Korea	Retrospective study	January 2000 to December 2010	230	CR group: 10 men and 133 women PS group: 5 men and 82 women	CR group: 69.6 years PS group: 68.3 years	253	159	94	10 and 15 years	-10 years: 95.4% - 15 years: 93.3%	-10 years: 92.7% - 15 years: 90.9%
Mayne et al. 2017 [20]	UK	Cohort study	March 1996 to April 2001	214	112 men 102 women	CR group: 70.6 years PS group: 70.8 years	214	107	107	10 years	99%	94.3%
Rand et al. 2003 [21]	USA	Retrospective study	January 1978 to December 2000	8290	3701 men 4589 women	69	11,606	8052	2994	10 years	91%	76%
Sartawi et al. 2018 [22]	USA	Retrospective double cohort study	July 1995 to July 1997	99	29 men 70 women	64	121	75	46	15 years	100%	98%
Serna-Berna et al. 2018 [3]	Spain	retrospective case–control study	2001 to 2006	479	CR group: 72 men and 196 women PS group: 67 men and 144 women	CR group: 68.8 years PS group: 70.1 years	479	268	211	15 years	95.7%	92.7%

### Results of the quality assessment of the included studies

**Fig. 2:**
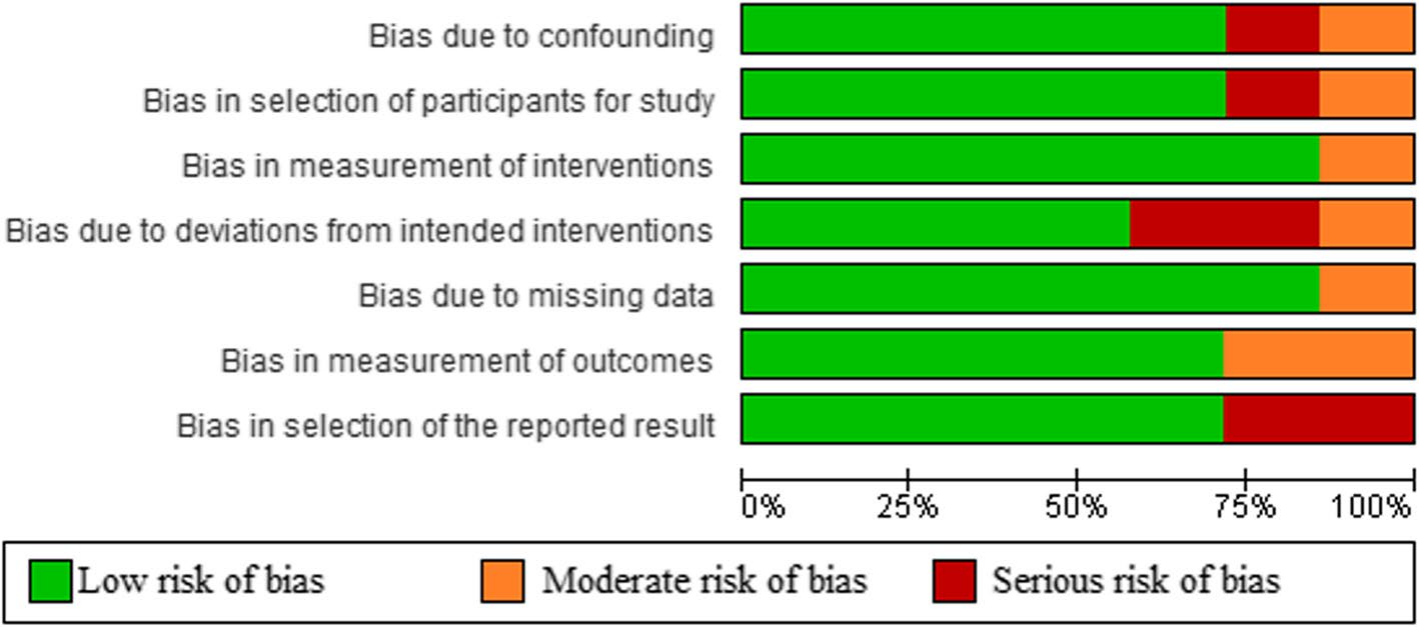
Risk of bias graph: review authors' judgements about each risk of bias item presented as percentages across all included studies

**Fig. 3:**
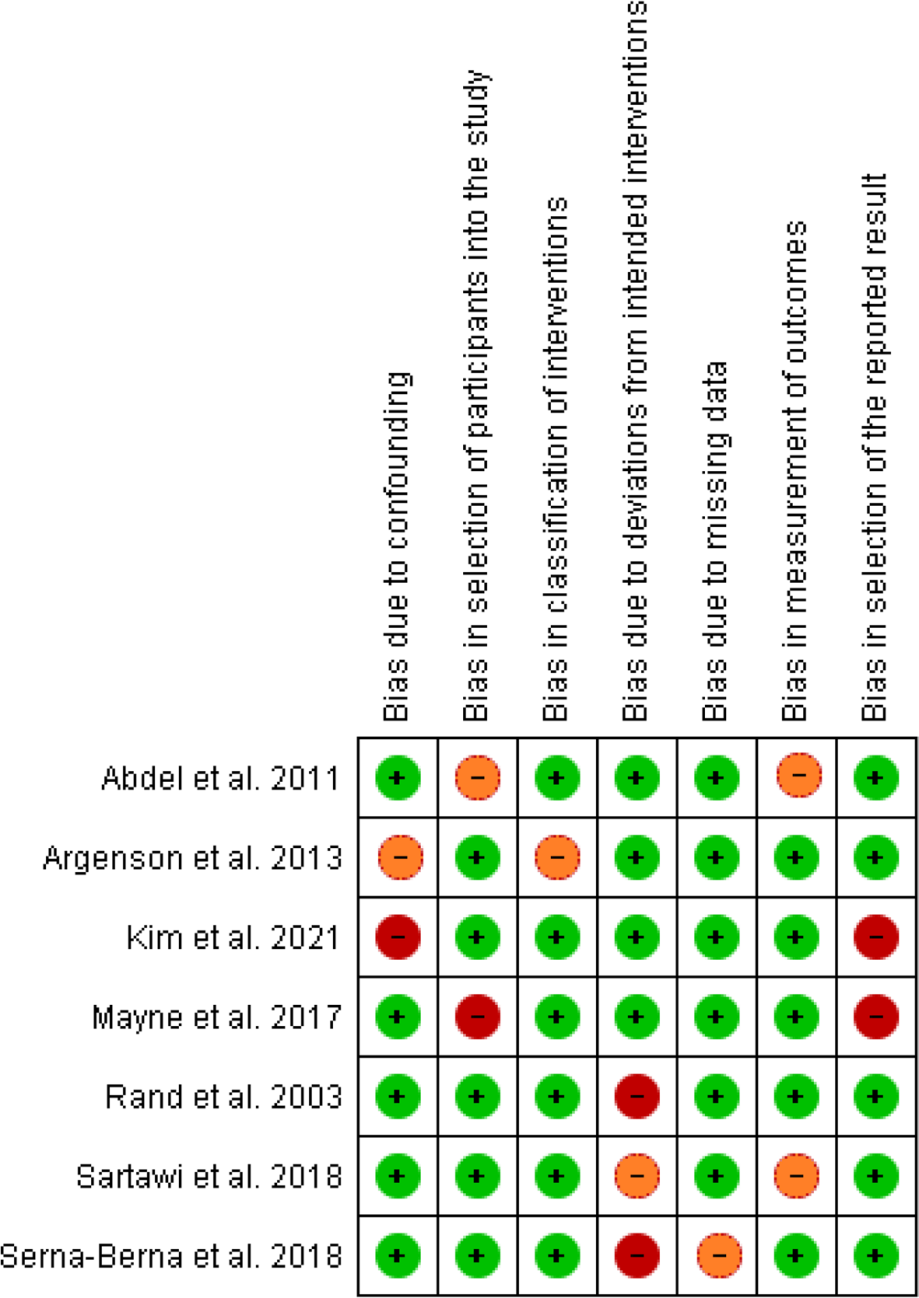
Risk of bias summary: review authors' judgements about each risk of bias item for each included study

Among observational studies, the majority of studies (6/7) presented a low risk of bias regarding the measurement of interventions and missing data, while 5/7 of studies presented a low risk of bias in confounding, selection of participants, measurement of outcomes and selection of the reported results. Regarding the deviations from intended interventions, 4 studies presented low risk while 2 and one studies showed serious and moderate risk, respectively (Figs. 2 and 3).

### Results of the analysis on survival

**Fig. 4:**
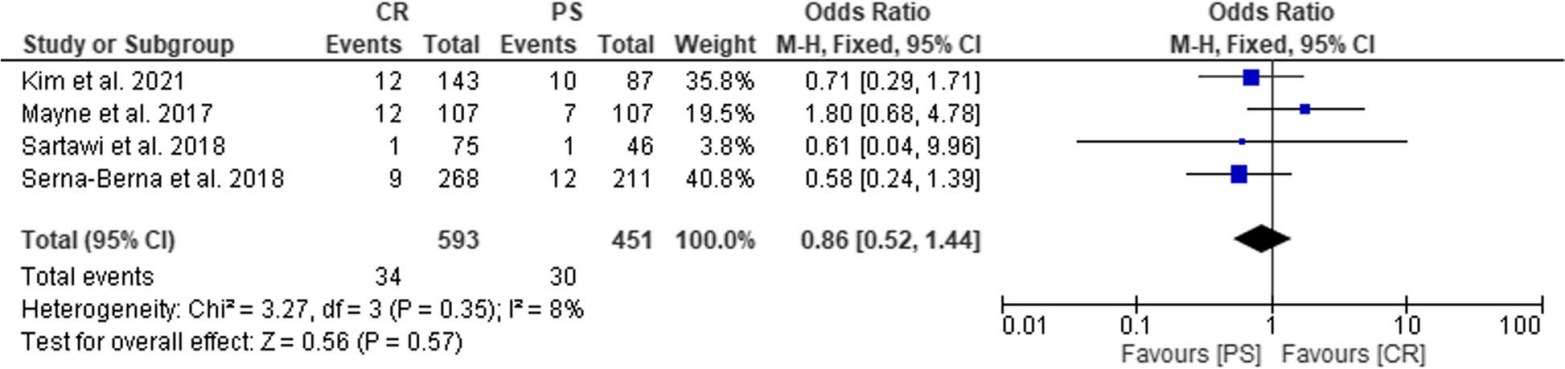
Forest plot representing the Odds Ratio of survivorship in CR and PS groups

The forest plot showed that survivorship outcome was significantly different between CR and PS groups. Indeed, CR had significantly better survival than PS (OR = 2.17; 95% CI: 1.69–2.80; *P* < 0.00001), but a high heterogeneity was detected (Chi^2^: 38.77, *P* < 0.00001, I^2^ = 77%) (Fig. 4).

### Results of the analysis on complications

**Fig. 5:**
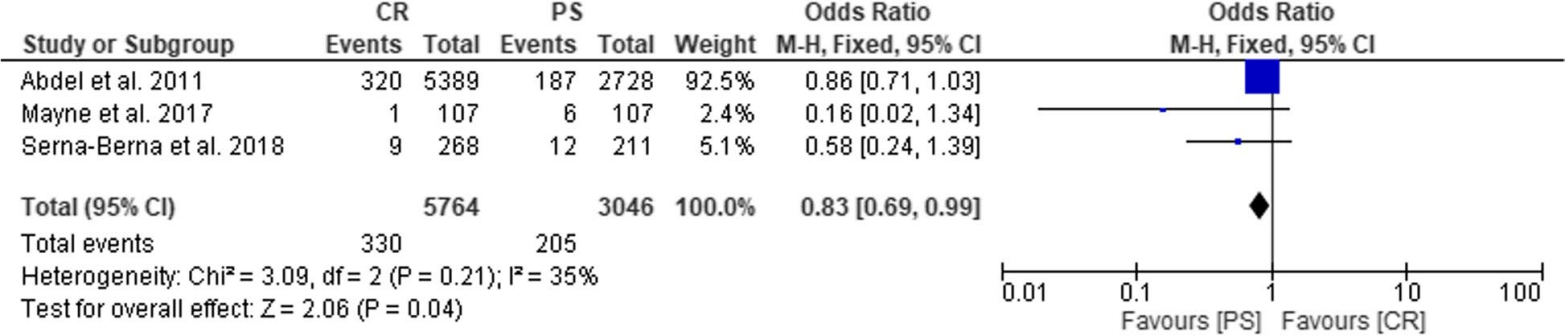
Forest plot representing the Odds Ratio of complication rate in CR and PS groups

The heterogeneity (Chi^2^ = 3.27, *P* = 0.35, I^2^ = 8%) was low, so a fixed effect model was used. The forest plot analysis showed that complication rate was not significantly different between CR and PS groups (OR = 0.86; 95% CI: 0.52–1.44; *P* = 0.57) (Fig. 5).

**Fig. 6:**
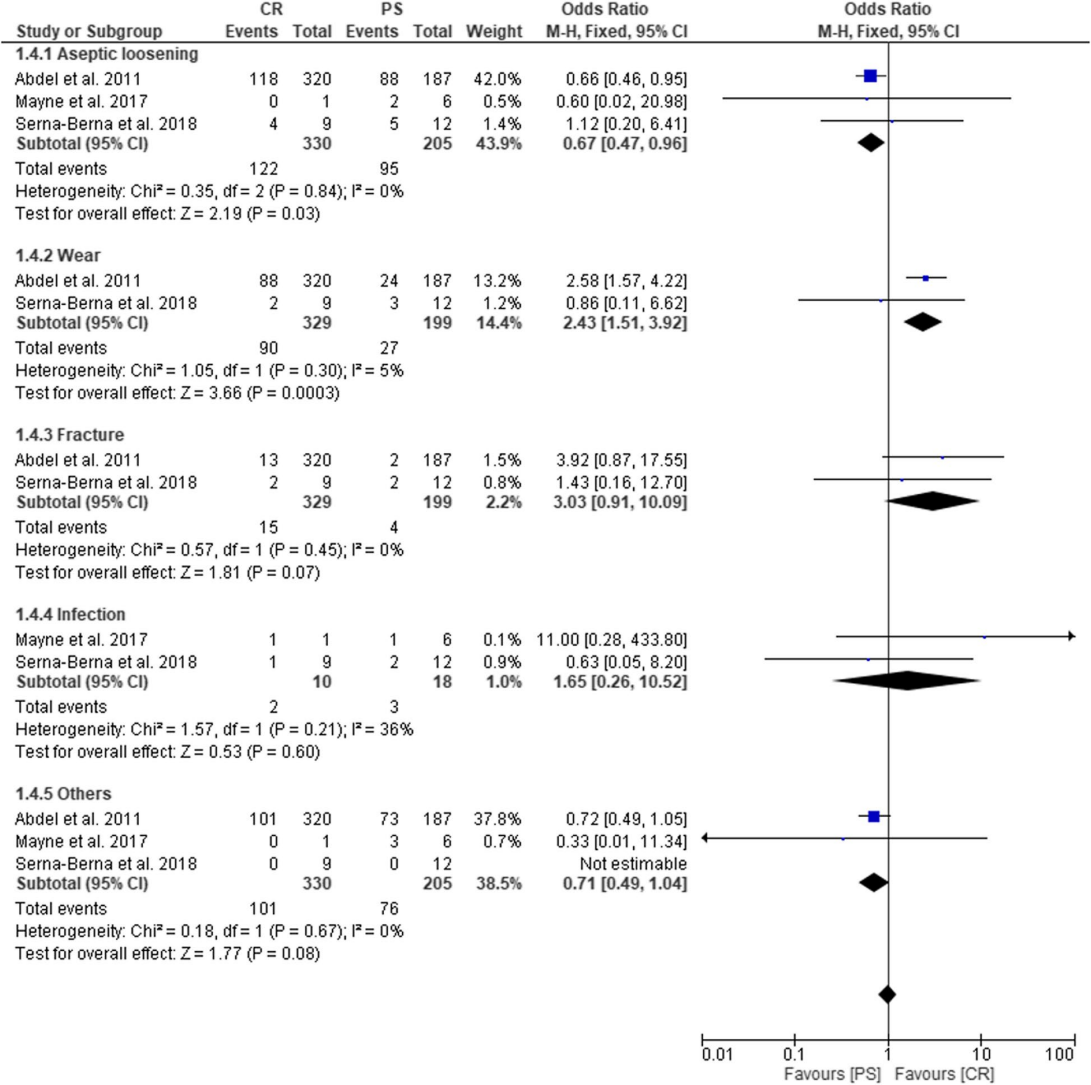
Forest plot representing the Odds Ratio of failure rate in CR and PS groups

Results of the analysis on failure The forest plot analysis revealed a low heterogeneity among studies (Chi^2^ = 3.09, *P* = 0.21, I^2^ = 35%) and showed that failure outcome was significantly different between CR and PS groups. Indeed, PS group had higher risk to present failure than CR group (OR = 0.83; 95% CI: 0.69–0.99; *P* = 0.04) (Fig. 6). The main causes of failure were aseptic loosening, followed by wear, fracture and infections. The forest plot analysis showed that the causes of failure were significantly different between CR and PS groups and the latter group had higher risk to present aseptic loosening (*P* < 0.05). However, infection did not differ significantly between CR and PS groups (*P* = 0.60) .

### Results of subgroup analysis

We carried out a subgroup analysis for survivorship outcome. The remaining outcomes were excluded given that the number of articles is limited According to the geographic origin, the highest OR of survivorship outcome was detected in studies published in USA (OR = 2.68, *P* < 0.0001), while European and Asian countries showed approximately similar OR (OR = 1.49 and OR = 1.68, respectively). However, heterogeneity between continents was not significant (Chi^2^ = 3.15, *P* = 0.21, I^2^ = 36.4%). When the period of publication was adopted as a moderator, OR significantly differed between studies published before and after 2011 (Chi^2^ = 4.64, *P* = 0.03, I^2^ = 78.4%). Indeed, the OR of survivorship outcome was higher in studies published before 2011 (OR = 2.67, *P* < 0.0001) than those published after 2011 (OR = 1.53, *P* = 0.05), likely due to shorter follow up period. Furthermore, the OR of survivorship outcome was approximately similar between 10 years (OR = 2.22, *P* = 0.0002) and 15 years (OR = 2.48, *P* < 0.0001) follow-up period (Chi^2^ = 0.24, *P* = 0.63, I^2^ = 0%). We concluded that only the period of publication constituted a source of heterogeneity in survivorship outcome (Table 2).

**Table 2:** Subgroup analysis of OR in terms of survivorship outcome

Subgroups	No. of studies	Odds Ratio (95% confidence interval)	*P*-value	Test for subgroup analysis
Geographic origin				
Europe	3	1.49 (0.70–3.14)	0.30	Chi^2^ = 3.15, *P* = 0.21, I^2^ = 36.4%
America	3	2.68 (2.06–3.48)	< 0.0001	
Asia	1	1.68 (0.80–3.52)	0.17	
Period of publication				
Before 2011	2	2.67 (2.05–3.48)	< 0.0001	Chi^2^ = 4.64, *P* = 0.03, I^2^ = 78.4%
After 2011	5	1.53 (0.99–2.36)	0.05	
Survival follow-up period				
10 years	5	2.22 (1.46–3.39)	0.0002	Chi^2^ = 0.24, *P* = 0.63, I^2^ = 0%
15 years	4	2.48 (2.19–2.81)	< 0.0001	

### Results of sensitivity analysis

Based on sensitivity analysis, we revealed that the outcome did not differ markedly, which indicates that the meta-analysis had strong reliability. Indeed, we revealed that the OR of survivorship outcome varied between (OR = 1.96, 95% CI: 1.35–2.86, *P* = 0.0004) and (OR = 2.36, 95% CI: 1.87–2.97, *P* < 0.00001) (Table 3).

**Table 3:** Sensitivity analyses of OR in terms of survivorship outcome

Study excluded	Odds Ratio (95% CI)	Heterogeneity
Abdel et al. 2011a	2.35 [1.82, 3.03], *P* < 0.00001	Chi^2^ = 22.25, (*P* = 0.04); I^2^ = 64%
Abdel et al. 2011b	1.96 [1.35, 2.86], *P* = 0.0004	Chi^2^ = 38.36, (*P* < 0.00001); I^2^ = 79%
Argenson et al. 2013c	2.36 [1.87, 2.97], *P* < 0.00001	Chi^2^ = 30.20, (*P* = 0.0002); I^2^ = 74%
Argenson et al. 2013d	2.18 [1.68, 2.82], *P* < 0.00001	Chi^2^ = 38.58, (*P* < 0.00001); I^2^ = 79%
Kim et al. 2021a	2.18 [1.68, 2.83], *P* < 0.00001	Chi^2^ = 38.52, (*P* < 0.00001); I^2^ = 79%
Kim et al. 2021b	2.22 [1.71, 2.88], *P* < 0.00001	Chi^2^ = 37.51, (*P* < 0.00001); I^2^ = 79%
Mayne et al. 2017	2.14 [1.66, 2.76], *P* < 0.00001	Chi^2^ = 38.11, (*P* < 0.00001); I^2^ = 79%
Rand et al. 2003	1.96 [1.51, 2.53], *P* < 0.00001	Chi^2^ = 17.09, (*P* = 0.03); I^2^ = 53%
Sartawi et al. 2018	2.16 [1.67, 2.79], *P* < 0.00001	Chi^2^ = 38.61, (*P* < 0.00001); I^2^ = 79%
Serna-Berna et al. 2018	2.22 [1.71, 2.89], *P* < 0.00001	Chi^2^ = .7.38, (*P* < 0.00001); I^2^ = 79%

## Discussion

The findings of this meta-analysis revealed a significantly superior survivorship of the CR group as compared to the PS group with aseptic revision surgery as the end point. Abdel et al. in a retrospective review of 8117 total knee arthroplasties performed over a 10 year period from 1988 to 1998 reported a 15 year survival of 90% for posterior cruciate retaining total knee arthroplasties, compared to 77% for posterior cruciate stabilizing total knee replacements [23]. Moreover, the superiority of the CR design was maintained even after accounting for factors like age, sex, diagnosis and preoperative deformity. Rand et al. analyzed patient and implant related factors affecting survivorship in 11,606 primary total knee arthroplasties [24]. They reported better survival rates with posterior cruciate retaining designs compared with posterior stabilized designs as early as five years after surgery with rates of 97% and 92% respectively. This difference was further amplified with 10-year survival rates of 91% and 76% respectively. The high heterogeneity seen in this meta-analysis is consistent with what is seen in literature. It may be attributed to the variability of available implant designs including the use of dished tibial components, constrained condylar implants, metal backed and all polyethylene components and the presence or absence of patella resurfacing [18] All of these could be potential sources of confounding. Furthermore, difference in surgical techniques and varied trends of clinical practice influencing patient selection in different geographical locations and the use of older generation of polyethylene with different sterilization techniques could also contribute to the same [25, 26]. These findings are consistent with what is seen in current literature and data from various Joint Replacement Registries. The Dutch Arthroplasty Register (van Steenbergen et al. 2015) had similar findings with higher midterm revision rates of posterior stabilized compared with cruciate retaining TKA [27]. This series included 133,841 cemented arthroplasties for osteoarthritis in the Netherlands from 2007–2016. The revision rates within 8 years of the primary procedure for males under 60 years of age for PS TKA systems was higher at 13% compared with 7.2% for CR TKA systems. However, such a significant difference was not seen in young females with revision rates of 9.4% and 8.2% for PS and CR TKA systems respectively. The most common reason for revision was found to be loosening of the tibial component, with a higher proportion in PS (41%) compared with a CR TKA system (27%). The second most common cause of revision was instability, with 36%in CR and 23% in PS knee systems. The post and cam design of PS TKA introduces higher constraint than CR design, which leads to increase stress at bone-implant interface and hence loosening. Data from the Australian Orthopaedic Association National Joint Replacement Registry also demonstrates a 45% higher risk of revision for the patients operated on by surgeons who prefer a posterior stabilized total knee replacement compared with those who prefer a minimally stabilized total knee replacement. Vertullo et al. in an analysis of 63,416 prosthesis from the registry between September 1999 to December 2014 observed a cumulative revision rate of 5% for surgeons who preferred the minimally stabilized replacement compared with 6% for those who preferred the posterior stabilized replacements [28]. The risks of revision was higher in the PS group for all causes including loosening or lysis and infection. However, these higher revision rates were only evident in male patients. The New Zealand joint registry 2022 report revealed a significantly higher revision rate for the posterior stabilized design (0.61/ 100 component years) compared to 0.41 and 0.46 for cruciate retaining and minimally stabilized knees respectively [15, 27]. The 18th Annual report (2021) of the British National joint registry also reports significantly higher revision rates in PS knees when compared with CR knees across various implant manufacturers [14].

However, no significant difference between the complication rates of the CR and PS groups was noted in this meta-analysis. These findings are consistent with existing literature. Longo et al. in a systematic review comparing the outcome of posterior stabilized to cruciate retaining total knee arthroplasty reported an overall complication rate of 3.9% (213 out of 5407 knees). No statistical difference was found between the two procedures [29]. In terms of etiology of failure, aseptic loosening, wear, fracture and infection were noted to be most prevalent in that order. Argenson et al. reported that the risk of infection was found to be increased in sedentary patients, while that of mechanical complications like loosening was increased in more active patients [13]. In a multivariate analysis Abdel et al. revealed that an age of more than 70 years, female sex, and a diagnosis of inflammatory arthritis were all associated with significantly greater survival [23]. It is often argued that PS TKA is preferred over CR TKA in severely deformed knees or other complex cases with doubtful PCL sufficiency [3], which might lead to inferior survivorship of PS TKA. However, the number of PS TKA and CR TKA cases included in the study is not significantly different. Also, with increasing use of ultracongruent insert in PCL deficient knees, PS TKA is no more the only choice for surgeons who prefer CR femoral prosthesis.

With improving access to quality healthcare, increased awareness and higher patient expectations, a larger number of young patients are undergoing total knee replacement [30]. Hence the rates of revision surgery are expected to rise subsequently [20, 31]. This adds morbidity to the patient while piling on to the financial burden on the healthcare system [19]. A clear consensus on long term implant survivorship is of paramount importance in formulating treatment protocols and choosing the right implant especially in this younger subset of patients [32].

In our knowledge, this is the first meta-analysis to compare CR TKA and PS TKA survival over long-term (over 10 years). The strengths of this meta-analysis include a comprehensive search strategy with well-defined inclusion and exclusion criteria. We exclusively included studies which evaluated long term survival with a minimum follow up of 10 years and the longest follow up being 20 years. Both manual and bibliographical searches were carried out. The articles included in this study had a relatively large sample size with the total number of TKR being 14,189. The usage of both implant designs was fairly proportionate with 7539 (53%) CR and 6650 (47%) PS knees.

### Limitations

There were many limitations of this meta-analysis including constraints due to the level of evidence of the included studies. Multiple factors like variability in implant design, surgical technique, patient selection bias etc. might confound our findings.

## Conclusions

The long-term survivorship of the cruciate retaining prosthesis is superior compared to the posterior stabilized design which makes CR the preferred implant, especially in younger patients. No discernible statistical difference has been demonstrated for either prosthesis design in terms of complication rates and other clinical outcomes including range of motion. Surgeons should try to adopt CR TKA techniques and use CR femur prosthesis with or without ultra-congruent insert as long as a satisfactory ligament balance is achieved. Continued long term follow up of randomized controlled trials is recommended to add to the volume of evidence which can eventually guide clinical practice.
